# Bioremediation of heavy metal contamination: recent developments and future directions

**DOI:** 10.1038/s41598-026-58756-9

**Published:** 2026-06-22

**Authors:** Xianmang Xu, Maria Augustyniak, Debasis Chakrabarty, Abhiroop Chowdhury, Sundaram Gunasekaran

**Affiliations:** 1 Heze Branch, Shandong Technological Innovation Center for High-Value Development and Utilization of Bio-Health Food, Qilu University of Technology (Shandong Academy of Sciences), Heze, China; 2 Institute of Biology, Biotechnology and Environmental Protection, University of Silesia in Katowice, Bankowa 9, 40-007 Katowice, Poland; 3 CSIR-National Botanical Research Institute, Lucknow, India; 4 Jindal School of Environment and Sustainability, O.P. Jindal Global University, Sonipat, India; 5 Departments of Biological Systems Engineering, Food Science and Materials Science & Engineering, University of Wisconsin-Madison, Madison, USA

**Keywords:** Biogeochemistry, Environmental sciences

## Abstract

Heavy metal pollution has silently insinuated itself into the fabric of modern life – from the vegetables on our dinner plates and the tap water we drink to the cosmetics we use daily. This reality underscores global environmental and public health crises intensified by industrialization and urban expansion. Conventional physical and chemical remediation methods for heavy metals, while effective to a degree, often involve prohibitive costs and risk disrupting the delicate balance of the original ecosystem. Consequently, the search for green, sustainable, and economically viable remediation alternatives has become imperative. This special issue brings together nine cutting-edge research papers that explore recent advances in heavy metal pollution control and resource recovery from diverse angles – including microbial remediation, plant–microbe combined approaches, bioleaching for resource utilization, soil amendment applications, and the ecological toxicity of nanoparticles. Collectively, these studies offer theoretical insights and novel practical strategies to support the development of efficient and sustainable technologies for managing heavy metal contamination. These research results can pave the way for deeper investigation into the efficacy of the proposed remediation with the eventual aim of taking the science from the lab to the field.

## Introduction

Heavy metals and metalloids are of particular environmental concern due to their potential to exert toxic effects on humans and other organisms, including microorganisms, even at extremely low concentrations^1,2^. These elements enter the environment through natural processes (e.g., soil erosion, forest fires, volcanic activity) and anthropogenic activities (e.g., mining, electroplating, pesticide and fertilizer use, and related consumer product consumption)^3^. If contaminated areas are not promptly remediated, heavy metals will migrate into surrounding water bodies and soils, thereby posing exposure risks to humans through ingestion, inhalation, or dermal absorption^4^. In the rapidly developing and changing world, understanding the issue of heavy metal pollution, its extent, and remediation techniques is vital.

Screening highly tolerant microbial strains from contaminated environments forms the foundation of bioremediation research. In a landmark study conducted in Germany’s Saxon state, Mungunkhuyag et al.^5^ successfully isolated multiple heavy metal-metal-resistant microalgae from an ancient mining site^5^. Among these, *Chlorella vulgaris* RG1-4 and *Tetradesmus obliquus* Ehr33-9 demonstrated exceptional copper removal efficiency of 100 mg/g and 89.73 mg/g, respectively, while Lobochlamys segnis Ehr31-1 exhibited remarkable cadmium adsorption capacity of 93.17 mg/g. This discovery not only validates the adaptive evolution of microorganisms in chronically polluted environments but also provides valuable resources for developing efficient and cost-effective biosorbents.

Similarly, Akoijam and Joshi^6^ investigated arsenic-contaminated paddy fields in India’s Manipur region, demonstrating that two plant-promoting bacteria – *Bacillus paramycoides* TNCB-27 and *Pseudomonas shirazica* TNB-16 – significantly reduced arsenic accumulation in rice aboveground tissues^6^. SEM and TEM observations revealed that these bacteria exhibited morphological changes under arsenic stress, including cell elongation and cytoplasmic contraction, and that these stress responses were closely linked to their arsenic-fixing capacity. Thus, indigenous plant-promoting bacteria could be utilized to mitigate arsenic transport to grains, thereby enhancing food security.

Bhojiya et al.^7^ screened a novel zinc-tolerant bacterium, *Chitinophaga niastensis* HMR31, from an Indian zinc mining area^7^. This strain exhibited excellent adsorption capacity for multiple heavy metals, including Zn(II), Fe(II), and Pb(II). Under optimized conditions (pH 7, 35 °C, 60-minute contact time), it achieved a zinc adsorption efficiency of 85.7%. Additionally, the strain produced various bioactive compounds such as indole-3-acetic acid (IAA), iron carriers, and 1-Aminocyclopropane-1-carboxylate (ACC) deaminase, significantly alleviating zinc stress-induced growth inhibition in mung beans. This study demonstrates the broad application potential of dual-functional strains with “remediation and bioassistance” capabilities in ecological restoration of mining areas.

Chauhan et al.^8^ conducted metagenomic analyses on long-term heavy metal-contaminated soils from the Savannah River Station and Oak Ridge National Nature Reserve in the United States, revealing complex relationships between mercury forms and microbial community structures^8^. The study found that total mercury content was not directly correlated with bioavailable mercury, while bacterial diversity decreased with increasing mercury concentrations, whereas fungal communities remained relatively stable. Stress-response genes, membrane transport proteins, and genes related to phosphorus metabolism pathways were enriched in dominant bacterial phyla such as Pseudomonadota and Bacteroidota. The presence of these functional genes explains the adaptive survival strategies of native microbial communities under prolonged pollution stress.

With the surge in e-waste, recycling rare earth elements (REEs) and critical metals has become a crucial direction in resource recycling. Nadi et al.^9^ experimentally revealed the dissolution mechanism of REEs by biological sulfuric acid, identifying the formation of iron sulfate passivation layers as the key factor limiting leaching efficiency^9^. Heydarian et al.^10^ optimized conditions for recycling spent lithium-ion batteries, achieving high-efficiency recovery^10^. Through shrinkage core model analysis, they confirmed that diffusion control is the rate-limiting step in leaching. These two studies collectively demonstrate that biometallurgical technology offers significant environmental and economic benefits for e-waste resource utilization.

In soil remediation practices, biochar and glomus fungal secreted glomus-related soil proteins (GRSP) have garnered significant attention due to their strong adsorption capacity for heavy metals. Teng et al.^11^ conducted a global meta-analysis to systematically evaluate the effects of biochar on soil chemical properties and copper availability^11^. The results demonstrated that physicochemical properties of biochar, such as ash content, specific surface area, and oxygen-containing functional groups, directly influence its remediation efficacy, providing a scientific basis for the targeted preparation of high-performance biochar materials. Tu et al.^12^ quantified the impact of urbanization intensity on the heavy metal retention capacity of GRSP^12^. The study found that with increasing urbanization intensity, GRSP content decreased by 19 to 24%, and its contribution to the retention of lead, cadmium, and chromium was reduced by 1.98 to 3.35 times. GRSP exhibited the highest adsorption potential for copper (14.71 to 23.77%), but this function was weakened by the inhibition of arbuscular mycorrhizal (AMF) activity and changes in light conditions caused by urbanization. These findings suggest that in urban soil management, it is essential to protect microbial symbiotic systems to maintain soil self-purification capacity.

While focusing on remediation technologies, the ecological risks posed by novel pollutants cannot be overlooked. Sibiya et al.^13^ investigated the toxic effects of titanium dioxide nanoparticles (TiO_2_ NPs) on the freshwater mussel *Lamellidens marginalis*^13^. After seven days of exposure to 100 μg/L TiO_2_ NPs, the mussel exhibited a significant decline in filtration rate and total blood cell count, inhibition of energy metabolism (ETS activity), elevated activity of antioxidant enzymes such as superoxide dismutase (SOD), catalase (CAT), and glutathione peroxidase (GPx), and a marked increase in lipid peroxidation levels. Histopathological analysis revealed severe damage to the gill tissue, including shedding of ciliated epithelial cells and disruption of intercellular junctions. These results indicate that even environmental concentrations of TiO_2_ NPs may impose multi-level physiological stress on benthic filter-feeding organisms.

Bioremediation of heavy metal pollution has developed a diversified strategy system: resistant microorganisms can directly adsorb heavy metals or indirectly leach them through metabolic products; the combination of plant growth-promoting rhizobacteria (PGPR) and plants can reduce the transfer of heavy metals to edible parts; biochar can passivate heavy metal activity by regulating soil physicochemical properties; GRSP plays a significant adsorption role in urbanized soils. However, heavy metal stress may co-screen antibiotic resistance genes, and nanomaterials themselves may pose ecological and human health risks.

Future research could usefully focus on deepening mechanistic understanding through integrated multi-omics and synthetic biology to explore heavy metal behavior in microbial-plant-soil systems, with AI-assisted screening and predictive modeling helping to optimize remediation materials and processes. Investigations into synergistic technologies are worth pursuing, such as combining hyperaccumulators with functional microbes and modified biochar, while addressing the challenges of scaling up from the laboratory to the field through long-term monitoring and considering circular-economy approaches, such as recovering metals from biomass. These technological efforts should be accompanied by ecological safety assessments that account for combined pollution with emerging contaminants, helping to ensure that remediation remains genuinely green. Cross-regional policy coordination, standardized protocols, and the potential inclusion of bioremediation technologies in national pollution control frameworks could further facilitate the translation of research into wider practice. Future research needs to focus on the policy instruments as well as the scalability of remediation technologies for wider application in polluted spaces.
